# Application of Deep Learning in Clinical Settings for Detecting and Classifying Malaria Parasites in Thin Blood Smears

**DOI:** 10.1093/ofid/ofad469

**Published:** 2023-09-15

**Authors:** Geng Wang, Guoju Luo, Heqing Lian, Lei Chen, Wei Wu, Hui Liu

**Affiliations:** Department of Clinical Laboratory, Peking Union Medical College Hospital, Beijing, China; Department of Clinical Laboratory, Peking Union Medical College Hospital, Beijing, China; Beijing Xiaoying Technology Co, Ltd, Beijing, China; Beijing Xiaoying Technology Co, Ltd, Beijing, China; Department of Clinical Laboratory, Peking Union Medical College Hospital, Beijing, China; Central Laboratory, Yunnan Institute of Parasite Diseases, Puer, China

**Keywords:** deep learning algorithm, malaria parasite, plasmodium, thin blood smear, YOLOv7

## Abstract

**Background:**

Scarcity of annotated image data sets of thin blood smears makes expert-level differentiation among *Plasmodium* species challenging. Here, we aimed to establish a deep learning algorithm for identifying and classifying malaria parasites in thin blood smears and evaluate its performance and clinical prospect.

**Methods:**

You Only Look Once v7 was used as the backbone network for training the artificial intelligence algorithm model. The training, validation, and test sets for each malaria parasite category were randomly selected. A comprehensive analysis was performed on 12 708 thin blood smear images of various infective stages of 12 546 malaria parasites, including *P falciparum*, *P vivax*, *P malariae*, *P ovale*, *P knowlesi*, and *P cynomolgi*. Peripheral blood samples were obtained from 380 patients diagnosed with malaria. Additionally, blood samples from monkeys diagnosed with malaria were used to analyze *P cynomolgi*. The accuracy for detecting *Plasmodium*-infected blood cells was assessed through various evaluation metrics.

**Results:**

The total time to identify 1116 malaria parasites was 13 seconds, with an average analysis time of 0.01 seconds for each parasite in the test set. The average precision was 0.902, with a recall and precision of infected erythrocytes of 96.0% and 94.9%, respectively. Sensitivity and specificity exceeded 96.8% and 99.3%, with an area under the receiver operating characteristic curve >0.999. The highest sensitivity (97.8%) and specificity (99.8%) were observed for trophozoites and merozoites.

**Conclusions:**

The algorithm can help facilitate the clinical and morphologic examination of malaria parasites.

## Teaser

A deep learning algorithm based on YOLOv7 achieved high accuracy (average precision, 0.902) in identifying and classifying *Plasmodium* species in thin blood smears. Sensitivity and specificity exceeded 96.8% and 99.3%, respectively, thus enabling improved clinical and morphologic examination of malaria parasites.

Malaria is a mosquito-borne infectious disease caused by *Plasmodium* species. According to the World Health Organization, >247 million malaria infection cases and 619 000 associated deaths occur worldwide [1]. The common human-infecting malaria parasites include *Plasmodium falciparum*, *P vivax*, *P malariae*, *P ovale*, *P knowlesi*, and *P cynomolgi* [2]. *Plasmodium* parasites proliferate in human red blood cells (RBCs) after infection, causing periodic systemic chills, fever, and sweating [3]. *P falciparum* infection can develop into fatal cerebral malaria or involve morbidity of multiple organs, such as acute renal failure and respiratory distress syndrome [4, 5]. However, early diagnosis and timely treatment can effectively reduce mortality [6].

*Plasmodium* infection is diagnosed by microscopic examination of peripheral blood smears, polymerase chain reaction (PCR) [7], and antigen-detecting rapid diagnostic tests [8]. PCR is time-consuming and expensive and requires sensitive equipment. Meanwhile, rapid diagnostic tests are less sensitive and susceptible to cross reaction, which occurs with other infectious disease parasites that share similar antigens with *Plasmodium*, yielding false-positive results [9].

The Centers for Disease Control and Prevention has established microscopic examination of Wright-Giemsa–stained thick and thin blood smears as the gold standard for malaria diagnosis [2]. Thick blood smears are used to assess the density of malaria parasites, whereas thin blood smears are used to diagnose malaria and identify all parasite stages and *Plasmodium* species [10]. The severity of malaria infection can be determined by the percentage of *Plasmodium*-infected RBCs, and the antimalarial treatment effect can be simultaneously monitored. However, the reliability of microscopic examination for diagnosing malaria depends largely on experienced morphologic experts and is thus subjective and time-consuming. Furthermore, it is difficult to standardize the microscopic examination of malaria parasites, especially in developing nations with limited experienced parasitologists [11].

Machine learning and automatic image recognition technologies have recently seen increasing application in disease diagnosis [12–15]. Implementing an automatic image analysis system based on deep learning can potentially mitigate the reliance on professional morphologists in the field of microscopy, thus reducing the time demands for clinical laboratory personnel and enhancing the efficiency and accuracy of malaria diagnosis. You Only Look Once v7 (YOLOv7) is a cutting-edge object detection system that employs a unified neural network architecture to perform object detection and classification in real time. Its ability to rapidly and accurately identify objects in images renders it particularly suitable for malaria diagnosis. However, to date, few studies have reported the use of automated image-processing algorithms for *Plasmodium* detection based on deep learning, and most of these studies relied on public data sets [16–27]. To our knowledge, there are no reports on the classification and staging of the 6 *Plasmodium* species detected in thin blood films from multicenter clinical samples according to the YOLOv7 algorithm.

Therefore, in this study, we aimed to establish a cutting-edge deep learning algorithm for the identification of *Plasmodium* species and their development stages in thin blood smears, evaluate its efficacy in augmenting microscopic examinations, and explore its potential clinical applications.

## METHODS

### Collection and Labeling of Microscopic Images

A total of 380 peripheral blood samples in dipotassium EDTA–containing tubes were obtained from patients diagnosed with malaria at the Peking Union Medical College Hospital and Yunnan Institute of Parasite Diseases from 2009 to 2021 (for patient distribution, see Supplementary Table 1). Blood samples of patients with >1 blood-borne infection were excluded. In addition, a single peripheral blood sample in a dipotassium EDTA–containing tube was obtained from a monkey diagnosed with *P cynomolgi* infection. Wright-Giemsa–stained thin blood smears were prepared according to the standardized procedure [2]. This study was approved by the Ethics Committee of the Peking Union Medical College Hospital (I-22PJ266). Written informed consent was obtained from all patients. A microscope (CX31; Olympus) and camera (acA1920; Basler) were used to capture 12 708 images (1920 × 1200 pixel resolution). The computer hardware configuration consisted of an Intel I9-9900 K CPU with 32GB RAM and an Nvidia RTX 2080Ti GPU. The operating system was Ubuntu 18.04 (Canonical), the technical framework was PyTorch 1.3 (Meta AI), and the functional code was written in Python 3.5. *Plasmodium* species were identified with real-time fluorescent PCR and nested PCR methods (for details of analysis steps, see Supplementary Text 1). Malaria parasites were labeled by 2 experienced parasitologists and reviewed by a third expert. Discrepancies were resolved by a fourth expert.

### Segmentation and Feature Extraction

**Figure 1.**
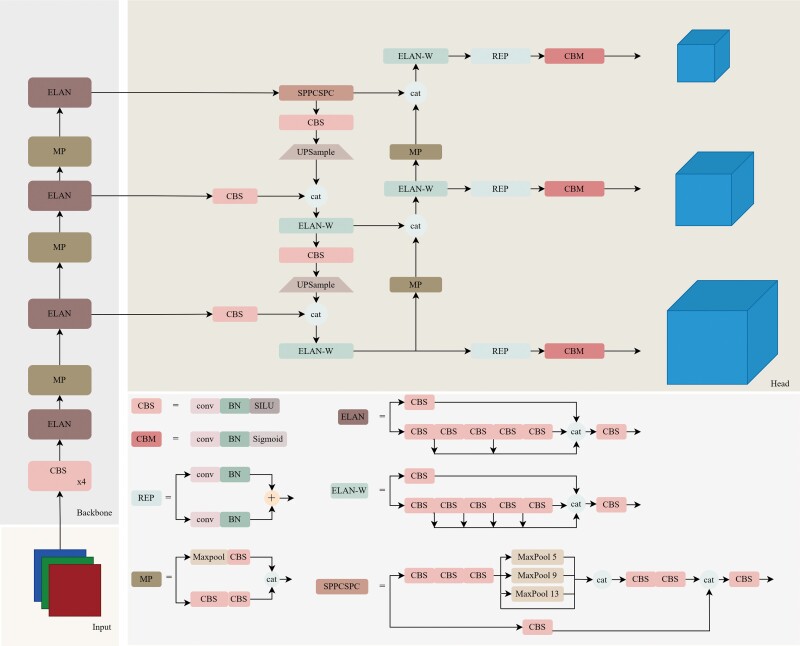
You Only Look Once v7 algorithm framework. BN, batch normalization; CBS, convolution batch normalization silu; CBM, convolution batch normalization mish; CSP, cross stage partial; ELAN, efficient layer aggregation network; MP, max pooling; REP, reparameterization; SPPCSPC, spatial pyramid pooling cross stage partial networks.

The YOLOv7 algorithm was adopted to complete the detection and recognition of *Plasmodium* through a 1-stage strategy for segmentation and feature extraction (ie, both were completed in 1 step). The algorithm framework is shown in Figure 1.

#### Backbone Network

The YOLOv7 algorithm adopted the Cross Stage Partial Network as the backbone network (Supplementary Figure 1). The amount of calculation was reduced by 20% as compared with that of the original network while still ensuring accuracy. This network thus achieved a suitable balance of accuracy and speed, with better application value.

The Cross Stage Partial Network removed the bottleneck layer (ie, the 1 × 1 convolution layer commonly used in ResNext and other networks) to avoid repeated gradient conduction and useless parameter calculation. The feature map was divided into feature maps A and B, according to the channel dimensions. Feature map A was convoluted and transformed into feature map A″; feature maps A″ and B were fused and transformed into cross stage partial (CSP) blocks; and each block was calculated similarly.

#### Feature Neck Layer

To improve small-target detection, YOLOv7 employed the Path Aggregation Network (PAN) algorithm for multiscale detection and feature fusion. The shallow feature map contained accurate location information conducive to network positioning, whereas the deep feature map contained rich high-level semantic information conducive to network classification; the combination of both maps can yield accurate positioning and classification features. The PAN algorithm deepened the feature path based on the feature pyramid network, thereby ensuring that the final classification and regression features contained high-precision location information and rich high-level semantic information to obtain more accurate positioning and classification results (Supplementary Figure 2).

#### Structural Reparameterization

The network structure based on “residual connections” had a high recognition accuracy; however, this was offset by its high memory and video memory consumption during inference, thereby reducing the inference speed and affecting the practical value of the model. Therefore, the structural reparameterization method exploited the “additivity” property of the convolution operation as follows:


$$con(x,w^{1})+con(x,w^{2})+con(x,w^{3})=con(x,w^{1}+w^{2}+w^{3}),$$

where *w*^1^, *w*^2^, and *w*^3^ were of the same size and the 1 × 1 convolution was treated as a 3 × 3 convolution with all positions except the center point set to 0. This ensured high recognition accuracy, considerably reduced the time required for inference, and maximized the performance of the model in terms of speed and accuracy (Supplementary Figure 3).

#### Prediction Layer

The PAN-generated feature map was used for multiscale prediction. The feature map contained 3 scales, with 3 anchor boxes at each feature point in each scale. Each anchor box outputted a vector of size: (confidence + detection frame coordinates) + number of categories. Each feature map outputted 64 × 64 × 3, 31 × 31 × 3, and 15 × 15 × 3 detection targets, resulting in 5282 detection results.

#### Nonmaximum Suppression

To suppress redundancy and low-quality test results, the nonmaximum suppression algorithm was used to deduplicate the test results. The nonmaximum suppression algorithm sorted the detection results by category according to the confidence level and calculated the intersection-over-union ratio among all detection frames in each category. The detection frame with the highest confidence was used as the final classification and segmentation result.

### Distribution of Images

In total, 12 708 thin blood smear images were obtained and analyzed, comprising 12 546 malaria-infected RBCs. The obtained images contained the following parasites: 4405 *P falciparum*, 3030 *P vivax*, 225 *P malariae*, 117 *P ovale*, 4630 *P knowlesi*, and 139 *P cynomolgi* at various stages, comprising 1776 trophozoites, 9285 at the ring stage, 1096 gametocytes, and 389 schizonts (Table 1).

**Table 1.** Distribution of *Plasmodium* Parasite Species

	Trophozoite	Ring	Gametocyte	Schizont	Total
*P falciparum*	3	3880	496	26	4405
*P vivax*	1161	1712	143	14	3030
*P malariae*	116	25	62	22	225
*P ovale*	62	10	42	3	117
*P knowlesi*	321	3648	346	315	4630
*P cynomolgi*	113	10	7	9	139
Total	1776	9285	1096	389	12 546

### Model Training

The training, validation, and test sets used for the model were stratifiedly selected at a ratio of 8:1:1 for each parasite stage. The training process is shown in Supplementary Figure 4.

#### Data Preprocessing

Before use, the images were preprocessed via the following steps (Supplementary Text 2):

1. Median filtering and noise reduction processing
2. Up-and-down and left-and-right flips
3. Random lighting, brightness, contrast, and saturation changes
4. Image filtering
5. Gaussian white noise addition to the image
6. Image mosaic
7. Image mixup
8. Image cropping and scaling to 640 × 640 pixels
9. Image normalization: pixel values were standardized to a normal distribution with a mean of 0 and variance of 1, and NumPy was converted to tensor vectors.

#### Hyperparameter Settings

The initial learning rate was 1e-3, the weight decay was 1e-4, and α was 0.99. The learning rate decay method was cosine, and the batch_size was set to 12.

#### Model Training Set

Stochastic gradient descent was used as the optimizer to iterate and optimize the model, and the maximum number of training epochs was 300. The model weight with the highest accuracy in the validation set was saved as the candidate weight. Changes in recall rate, model accuracy, mean average precision (mAP), box loss, and confidence loss during model training were recorded.

## RESULTS

### Efficiency Analysis

**Figure 2.**
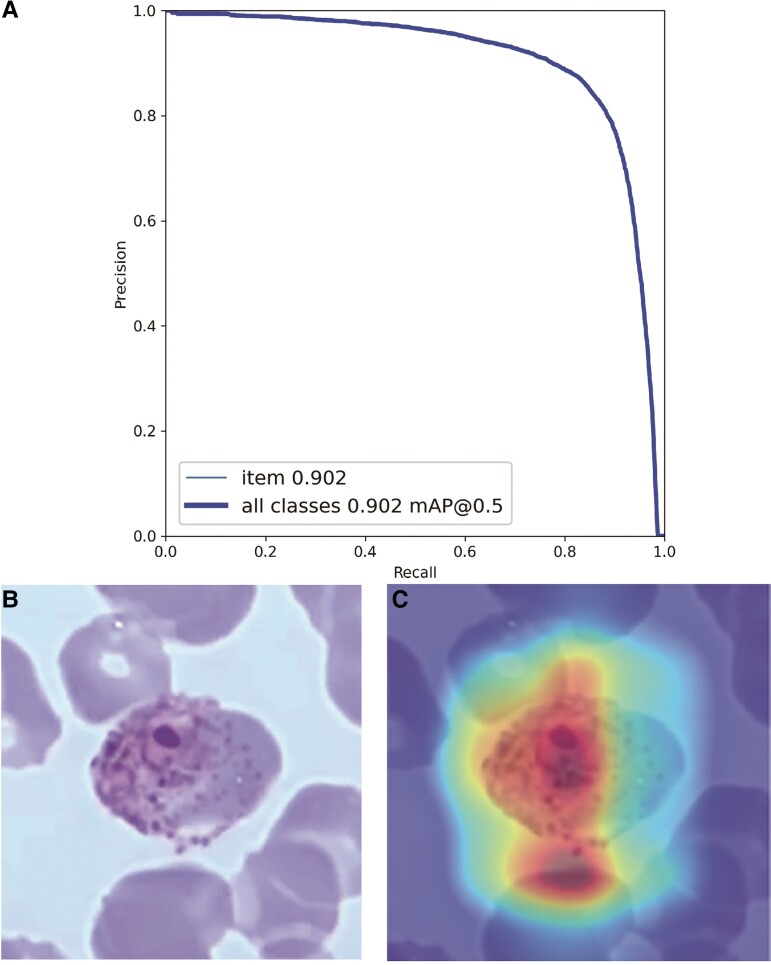
*A*, Precision-recall curve of the algorithm. Heat map for classifying malaria parasites: *B*, the original picture; *C*, the heat map. Red and blue depict high and low attention, respectively. mAP, mean average precision.

The total analysis time for 1116 parasites in 782 images was 13 seconds, and the average analysis time for a single image was 0.016 seconds. The average recognition time for a single parasite was 0.014 seconds, and mAP was 0.902. The precision-recall curve of the algorithm is shown in Figure 2*A*. Figure 2 shows the heat diagram for determining the malaria parasite stages: Figure 2*B* shows the original picture, and Figure 2*C* shows the attention of the neural network to determine parasite characteristics. Red and blue depict high and low attention, respectively. The algorithm exhibited a high level of attention to the actual characteristics of malaria parasites (Figure 2*B*).

#### Identification of Infected RBCs

**Figure 3.**
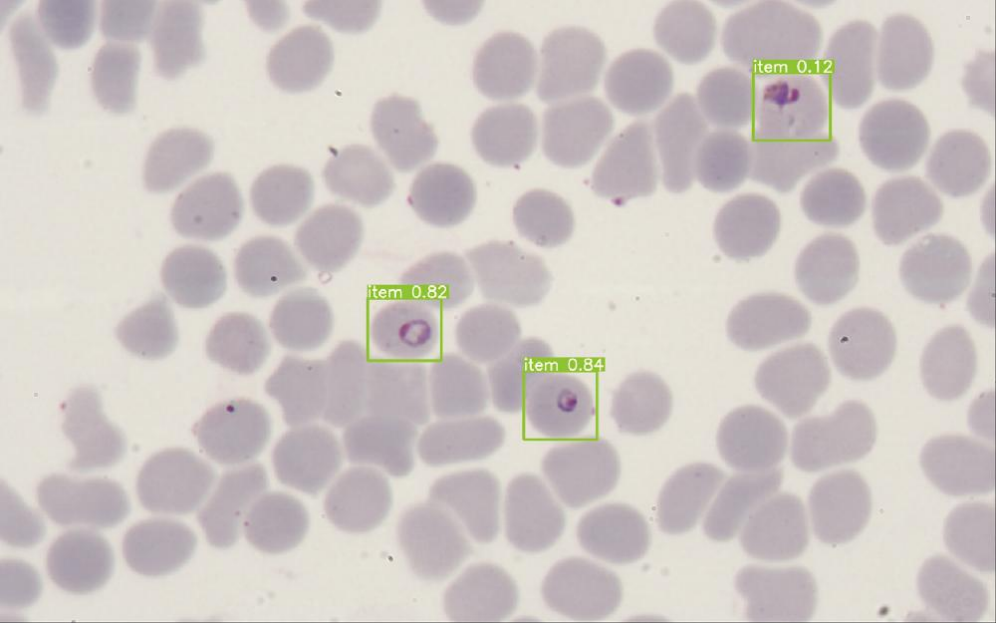
Output of the algorithm results. Boxes indicate *Plasmodium*.

For the 1116 parasites in the test set, the recall and precision of the ability to identify infected erythrocytes were 96.0% and 94.9%, respectively. Figure 3 shows an example of the output of the algorithm.

#### Classification and Staging of *Plasmodium*

**Figure 4.**
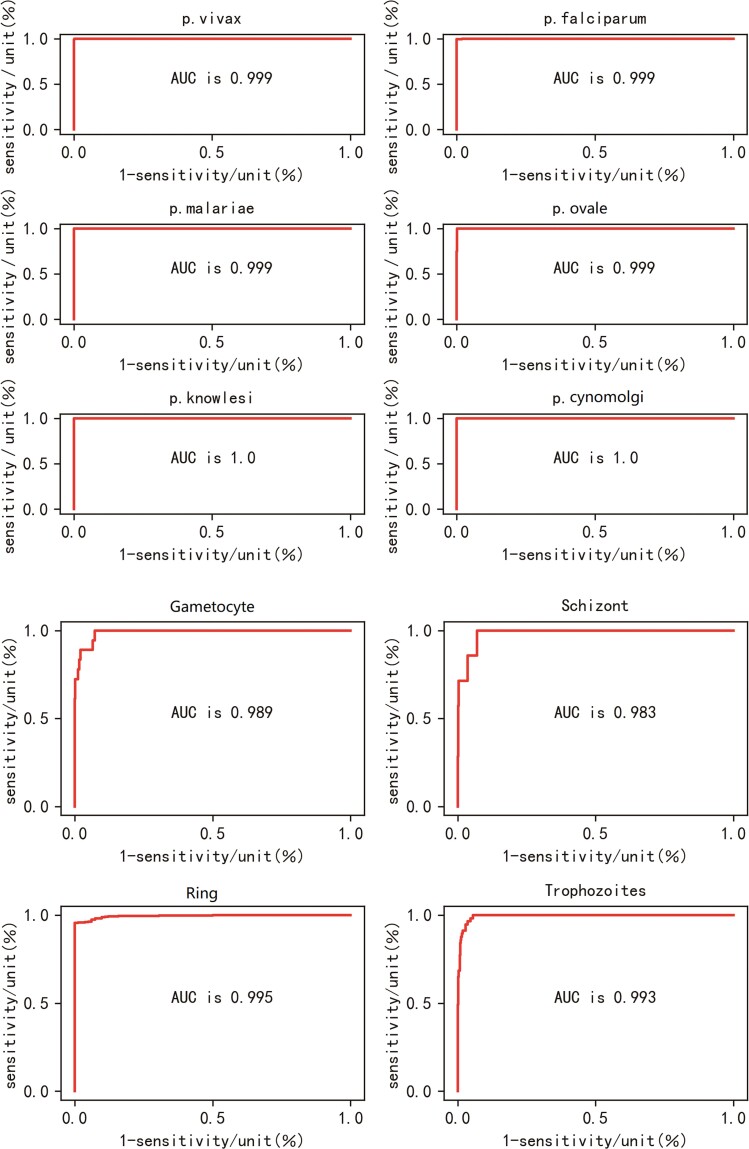
Receiver operating characteristic curves of the model for the different stages and classification of *Plasmodium*. AUC, area under the curve.

The overall sensitivity of the algorithm to parasites was 96.0% (for confusion matrix, see Supplementary Table 2). The performance of the algorithm in classifying and staging *Plasmodium* is summarized in Table 2, and the receiver operating characteristic curve is shown in Figure 4. The sensitivity and specificity of the algorithm for species classification were >96.8%, and the area under the curve (AUC) was >0.999. Among the 4 stages, the highest sensitivity (97.8%) and best specificity (99.8%) were observed for the ring stage and schizonts, respectively. The 4 stages had an AUC >0.983. An example of the output result of the algorithm is shown in Supplementary Figure 5.

**Table 2.** Performance of the Algorithm for *Plasmodium* Species Classification With PCR as the Reference Method

	Sensitivity	Specificity	False Positive	False Negative
*P falciparum*	96.80	99.30	0.20	3.2
*P vivax*	99.70	99.60	0.30	0.3
*P malariae*	99.80	99.80	0.10	0.2
*P ovale*	99.70	99.50	0.10	0.3
*P knowlesi*	99.90	99.90	0.40	0.1
*P cynomolgi*	99.90	99.90	0.10	0.1
Gametocyte	72.0	99.2	0.8	28
Schizont	85.7	99.8	0.2	14.3
Ring	97.8	84.9	15.1	2.2
Trophozoite	80.7	98.2	1.8	19.3

Data are presented as percentages.

Abbreviation: PCR, polymerase chain reaction.

## DISCUSSION

In this study, we developed an algorithm based on deep learning for *Plasmodium* species classification and staging in thin blood smears. The performance of the model was good; the scanning time of a single image was 0.016 seconds; and the mAP of the algorithm was 0.902. The recall and precision were 96.0% and 94.9%, respectively. The sensitivity and specificity for the 6 *Plasmodium* species were ≥96.8% and ≥99.3%. Thus, the algorithm can provide effective and accurate auxiliary diagnostic information for the clinical detection of *Plasmodium* in thin blood smears.

Timely and accurate detection of malaria parasites is essential for diagnosing and treating malaria. There are several approaches to malaria diagnosis, with microscopic examination representing the gold standard. However, malaria may be undetected in regions with inadequate medical resources because of limited professional capacity. Therefore, morphologic examination of parasitemia is currently a major challenge. With the gradual increase in the application of artificial intelligence (AI) in the medical field, deep learning algorithms can be used to detect malaria parasites [28]. Yet, parasite images in blood smears currently constitute a bottleneck for machine learning training [29]: several images must be labeled and annotated, which is time-consuming and laborious, and the method requires the participation of experienced parasite morphologists.

Previous studies have often used public data sets containing a single *Plasmodium* species (eg, *P vivax* on https://data.broadinstitute.org/bbbc/BBBC041/) or lacking image segmentation (eg, National Institutes of Health, https://lhncbc.nlm.nih.gov/publication/pub9932) for model training [16, 20, 24, 26, 27]. The performance of the model depends largely on the quality and extent of the training sets and the similarity between the validation and training images. The thin blood films in the present study were obtained from 2 centers and were labeled by experienced morphologic experts, ensuring the quality of the data set. Kuo et al [21] reported that the AUC of their established algorithms for identifying *P falciparum* in 8145 images from 36 thin blood smears was 0.997, with a specific AUC of 0.995 for the ring stage. The present study yielded comparable results; however, the established algorithm incorporated the identification of 6 *Plasmodium* species and achieved an mAP of 0.902, which was superior to the mAP (0.885) reported by Kuo et al. Furthermore, Sriporn et al [24] reported the accuracy, precision, and recall rates for identifying a single *Plasmodium* species in 7000 images from the public data of the National Library of Medicine to be within the ranges of 76.07% to 99.28%, 76.07% to 99.29%, and 78.05% to 99.28%, respectively. Yet, the duration of their process was substantially longer, from 14 to 125 minutes, and the scope of their analysis was limited to a single *Plasmodium* species. In the present study, the YOLOv7 algorithm was used as the primary technique for detecting *Plasmodium*. The average analysis time was 0.02 seconds per single field image with dimensions of 15 × 15 mm, which is considerably shorter than that reported by Sriporn et al (1.3–10 seconds per image). The analysis of 22 500 field images from thin blood smears resulted in an overall analysis time of approximately 7.5 minutes, demonstrating the efficacy of the YOLOv7 algorithm in reducing the analysis time. The key factor for the efficiency and accuracy of the YOLOv7 algorithm lies in its resolution of object detection as a regression problem, in which target region and target category predictions were integrated into a single neural network model. This approach enabled the direct transformation of the input image into output object locations and classifications, enhancing the detection speed and precision. In contrast, previous studies have mainly employed traditional image-processing techniques or slower 2-stage detectors, such as faster R-CNN [20, 30], VGG-16 [31], and SPPnet [32], which often result in long analysis time and low accuracy.

In the present study, 12 708 thin blood smear images with different parasite species and development stages were obtained from multicenter clinical settings. These images were labeled by experienced morphologic experts according to the criteria of the Centers for Disease Control and Prevention. There is a lack of studies on the accuracy of detecting and classifying the 6 *Plasmodium* species in thin blood smears included in this study. Our findings demonstrated that the proposed algorithm achieved a high level of recognition sensitivity and specificity for the 6 *Plasmodium* species tested, with values consistently exceeding 96.8% and 99.3%, respectively. Although 5 of these parasites (*P falciparum*, *P vivax*, *P malariae*, *P ovale*, and *P knowlesi*) are common human-infecting *Plasmodium* species, *P cynomolgi* can infect monkeys and infrequently humans; thus, we chose to include it in our analysis. Malaria parasites at different development stages have different biological and metabolic characteristics in the host and mosquito, with consequently different degrees of sensitivity and resistance to different types of drugs and treatments. Understanding the development stage of the malaria parasite can thus help clinicians select the most effective treatment plan and improve treatment outcomes [2]. Here, the YOLOv7 algorithm was performed efficiently and accurately, providing clinical technologists with a valuable tool for the microscopic examination of malaria parasites and the best standardization tool for identifying thin blood smears.

This study has some limitations. First, in the training and validation sets, the number of *P ovale* and schizont-stage *Plasmodium* species was small. Thus, future studies should include a larger number of training sets for these *Plasmodium* species to improve the accuracy of the algorithm. Moreover, the sample size of the training and validation sets for *Plasmodium* species other than *P falciparum* and the ring-stage *Plasmodium* species was limited. Second, these studies were performed in a reference laboratory, and additional studies are currently underway to test the approach in a clinical setting. In our future studies, we will focus on establishing an AI algorithm to analyze thick blood smear images, which will encompass parasite density calculation and contribute to a more advanced application of deep learning to support malaria diagnosis.

## CONCLUSIONS

In this study, we developed and evaluated an AI algorithm for identifying *Plasmodium* species in thin blood smears. The algorithm exhibited a high degree of sensitivity and specificity, indicating its potential as a valuable tool to aid clinical technologists in the microscopic detection of malaria parasites.

## Supplementary Material

ofad469_Supplementary_Data

## Supplementary Data

Supplementary materials are available at *Open Forum Infectious Diseases* online. Consisting of data provided by the authors to benefit the reader, the posted materials are not copyedited and are the sole responsibility of the authors, so questions or comments should be addressed to the corresponding author.

## Notes

***Author contributions.*** G. W., G. L., and W. W. have full access to all data used in the study and take responsibility for the integrity of the data and the accuracy of the data analysis. Concept and design: G. W., G. L., H. Lian, L. C., W. W., H. Liu. Image annotation: G. W., H. Liu, W. W. Drafting of the manuscript: G. L., H. Lian, W. W. Critical revision of the manuscript for important intellectual content: G. L. Statistical analysis: G. W., G. L., H. Lian, L. C. Technical algorithmic support: H. Lian, L. C.

***Availability of data and materials*.** Data are not publicly available. Owing to the nature of the study, the raw data that support the findings of this study cannot be shared with other researchers.

***Patient consent statement.*** Written informed consent was obtained from all patients.

***Financial support.*** This work was supported by the National High-Level Hospital Clinical Research Funding (2022-PUMCH-B-074); and Beijing Municipal Science and Technology Commission, Administrative Commission of Zhongguancun Science Park (Z221100003522004).

## References

[1] World Health Organization . World malaria report 2022. Geneva: World Health Organization, 2022.

[2] Centers for Disease Control and Prevention . Malaria diagnostic techniques. Available at: https://www.cdc.gov/malaria/diagnosis_treatment/diagnostic_tools.html. Accessed January 1, 2023.

[3] Walter K, John CC. Malaria. JAMA 2022; 327:597. 35133414 10.1001/jama.2021.21468

[4] Bartoloni A, Zammarchi L. Clinical aspects of uncomplicated and severe malaria. Mediterr J Hematol Infect Dis 2012; 4:e2012026. 22708041 10.4084/MJHID.2012.026 PMC3375727

[5] Word Health Organization . WHO recommends groundbreaking malaria vaccine for children at risk. Available at: https://www.who.int/news/item/06-10-2021-who-recommends-groundbreaking-malaria-vaccine-for-children-at-risk. Accessed January 1, 2023.

[6] Daily JP, Minuti A, Khan N. Diagnosis, treatment, and prevention of malaria in the US: a review. JAMA 2022; 328:460–71. 35916842 10.1001/jama.2022.12366

[7] Feleke DG, Alemu Y, Yemanebirhane N. Performance of rapid diagnostic tests, microscopy, loop-mediated isothermal amplification (LAMP) and PCR for malaria diagnosis in Ethiopia: a systematic review and meta-analysis. Malar J 2021; 20:384. 34579729 10.1186/s12936-021-03923-8 PMC8474705

[8] Ajakaye OG, Ibukunoluwa MR. Performance evaluation of a popular malaria RDT in Nigeria compared with microscopy. J Parasit Dis 2020; 44:122–5. 32174714 10.1007/s12639-019-01170-y PMC7046886

[9] Kavanaugh MJ, Azzam SE, Rockabrand DM. Malaria rapid diagnostic tests: literary review and recommendation for a quality assurance, quality control algorithm. Diagnostics (Basel) 2021; 11:768. 33922917 10.3390/diagnostics11050768 PMC8145891

[10] World Health Organization . Giemsa staining of malaria blood films: malaria microscopy standard operating procedure—MM-SOP-07A. Available at: http://www.wpro.who.int/mvp/lab_quality/2096_oms_gmp_sop_07a_rev.pdf. Accessed January 1, 2023.

[11] Varo R, Balanza N, Mayor A, Bassat Q. Diagnosis of clinical malaria in endemic settings. Expert Rev Anti Infect Ther 2021; 19:79–92. 32772759 10.1080/14787210.2020.1807940

[12] Alzubaidi L, Zhang J, Humaidi AJ, et al Review of deep learning: concepts, CNN architectures, challenges, applications, future directions. J Big Data 2021; 8:53. 33816053 10.1186/s40537-021-00444-8 PMC8010506

[13] Anwar SM, Majid M, Qayyum A, Awais M, Alnowami M, Khan MK. Medical image analysis using convolutional neural networks: a review. J Med Syst 2018; 42:226. 30298337 10.1007/s10916-018-1088-1

[14] Poostchi M, Silamut K, Maude RJ, Jaeger S, Thoma G. Image analysis and machine learning for detecting malaria. Transl Res 2018; 194:36–55. 29360430 10.1016/j.trsl.2017.12.004 PMC5840030

[15] Tiulpin A, Thevenot J, Rahtu E, Lehenkari P, Saarakkala S. Automatic knee osteoarthritis diagnosis from plain radiographs: a deep learning-based approach. Sci Rep 2018; 8:1727. 29379060 10.1038/s41598-018-20132-7 PMC5789045

[16] Abdurahman F, Fante KA, Aliy M. Malaria parasite detection in thick blood smear microscopic images using modified YOLOV3 and YOLOV4 models. BMC Bioinform 2021; 22:112. 10.1186/s12859-021-04036-4 PMC7938584 33685401

[17] Abubakar A, Ajuji M, Yahya IU. DeepFMD: computational analysis for malaria detection in blood-smear images using deep-learning features. Appl Syst Innov 2021; 4:82.

[18] Davidson MS, Andradi-Brown C, Yahiya S, et al Automated detection and staging of malaria parasites from cytological smears using convolutional neural networks. Biol Imaging 2021; 1:e2. 35036920 10.1017/S2633903X21000015 PMC8724263

[19] Gopakumar GP, Swetha M, Sai Siva G, Sai Subrahmanyam GRK. Convolutional neural network-based malaria diagnosis from focus stack of blood smear images acquired using custom-built slide scanner. J Biophotonics 2018; 11:10. 10.1002/jbio.201700003 28851134

[20] Hung J, Lopes SCP, Nery OA, et al Applying faster R-CNN for object detection on malaria images. Conf Comput Vis Pattern Recognit Workshops 2017; 2017:808–13. 34938593 10.1109/cvprw.2017.112 PMC8691760

[21] Kuo PC, Cheng HY, Chen PF, et al Assessment of expert-level automated detection of plasmodium falciparum in digitized thin blood smear images. JAMA Netw Open 2020; 3:e200206. 32108895 10.1001/jamanetworkopen.2020.0206 PMC7049085

[22] Manescu P, Shaw MJ, Elmi M, et al Expert-level automated malaria diagnosis on routine blood films with deep neural networks. Am J Hematol 2020; 95:883–91. 32282969 10.1002/ajh.25827

[23] Oliveira AD, Prats C, Espasa M, et al The malaria system microapp: a new, mobile device-based tool for malaria diagnosis. JMIR Res Protoc 2017; 6:e70. 28442456 10.2196/resprot.6758 PMC5424126

[24] Sriporn K, Tsai CF, Tsai CE, Wang P. Analyzing malaria disease using effective deep learning approach. Diagnostics (Basel) 2020; 10:744. 32987888 10.3390/diagnostics10100744 PMC7601431

[25] Yang F, Poostchi M, Yu H, et al Deep learning for smartphone-based malaria parasite detection in thick blood smears. IEEE J Biomed Health Inform 2020; 24:1427–38. 31545747 10.1109/JBHI.2019.2939121 PMC12077747

[26] Yu H, Yang F, Rajaraman S, et al Malaria screener: a smartphone application for automated malaria screening. BMC Infect Dis 2020; 20:825. 33176716 10.1186/s12879-020-05453-1 PMC7656677

[27] Zhao OS, Kolluri N, Anand A, et al Convolutional neural networks to automate the screening of malaria in low-resource countries. PeerJ 2020; 8:e9674. 32832279 10.7717/peerj.9674 PMC7413078

[28] Shambhu S, Koundal D, Das P, Hoang VT, Tran-Trung K, Turabieh H. Computational methods for automated analysis of malaria parasite using blood smear images: recent advances. Comput Intell Neurosci 2022; 2022:3626726. 35449742 10.1155/2022/3626726 PMC9017520

[29] Maturana CR, de Oliveira AD, Nadal S, et al Advances and challenges in automated malaria diagnosis using digital microscopy imaging with artificial intelligence tools: a review. Front Microbiol 2022; 13:1006659. 36458185 10.3389/fmicb.2022.1006659 PMC9705958

[30] Ren S, He K, Girshick R, Sun J, Faster R. Faster R-CNN: towards real-time object detection with region proposal networks. IEEE Trans Pattern Anal Mach Intell 2017; 39:1137–49. 27295650 10.1109/TPAMI.2016.2577031

[31] Molina A, Rodellar J, Boldú L, Acevedo A, Alférez S, Merino A. Automatic identification of malaria and other red blood cell inclusions using convolutional neural networks. Comput Biol Med 2021; 136:104680. 34329861 10.1016/j.compbiomed.2021.104680

[32] Zhou H, Wang Y, Ye M. A method of CNN traffic classification based on sppnet. In: 14th International Conference on Computational Intelligence and Security (CIS) 2018. New York: IEEE Publications, 2018:390–4. 10.1109/CIS2018.2018.00093.

