# Supplementary material for: Application of Deep Learning in Clinical Settings for Detecting and Classifying Malaria Parasites in Thin Blood Smears
Source: Open Forum Infect Dis. 2023 Sep 15;10(11):ofad469. doi: 10.1093/ofid/ofad469 (PMC10627339; doi:10.1093/ofid/ofad469)
Supplement: ofad469_Supplementary_Data [file ofad469_supplementary_data.docx]

Supplementary Material

Application of deep learning in clinical settings for detecting and classifying malaria parasites in thin blood smears

Geng Wang^1,†^, Guoju Luo^1,†^, Heqing Lian^3^, Lei Chen^3^, Wei Wu^1*^, Hui Liu^2*^

**^*^Correspondence to:**

Wei Wu, Department of Clinical Laboratory, Peking Union Medical College Hospital, Beijing 100730, China. Email: [ww_pumch@sina.com](mailto:ww_pumch@sina.com); Tel.: 86-10-69159710, Fax: +8613611245564

Hui Liu, Yunnan Institute of Parasite Diseases, Puer, 665000, China. Email: liubible @126.com;

Tel: 86-0879-2122153, Fax: +86188 1230 9887


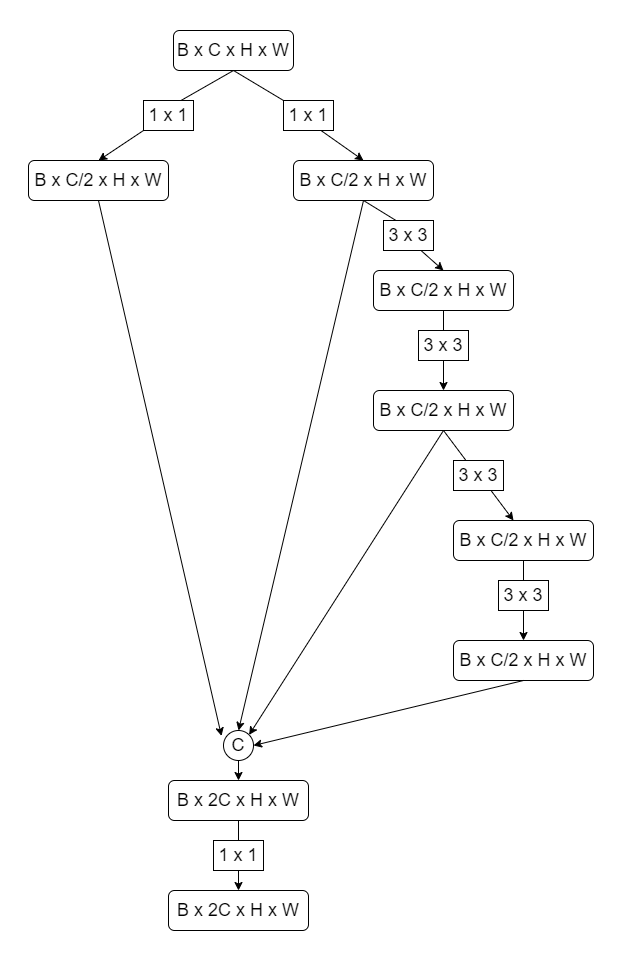


**Supplementary Figure 1.** Efficient layer aggregation network (ELAN) module


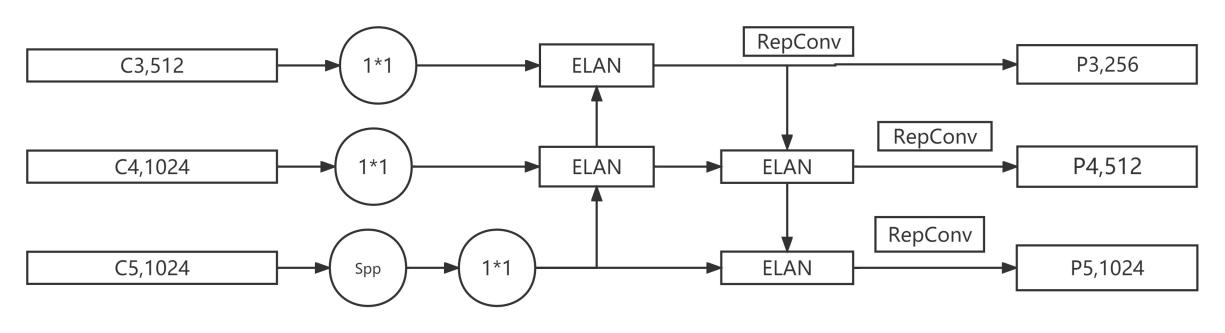


**Supplementary Figure 2.** Feature neck layer


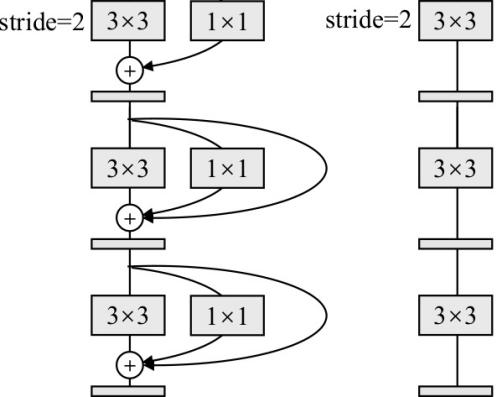


**Supplementary Figure 3.** Structural re-parameterization


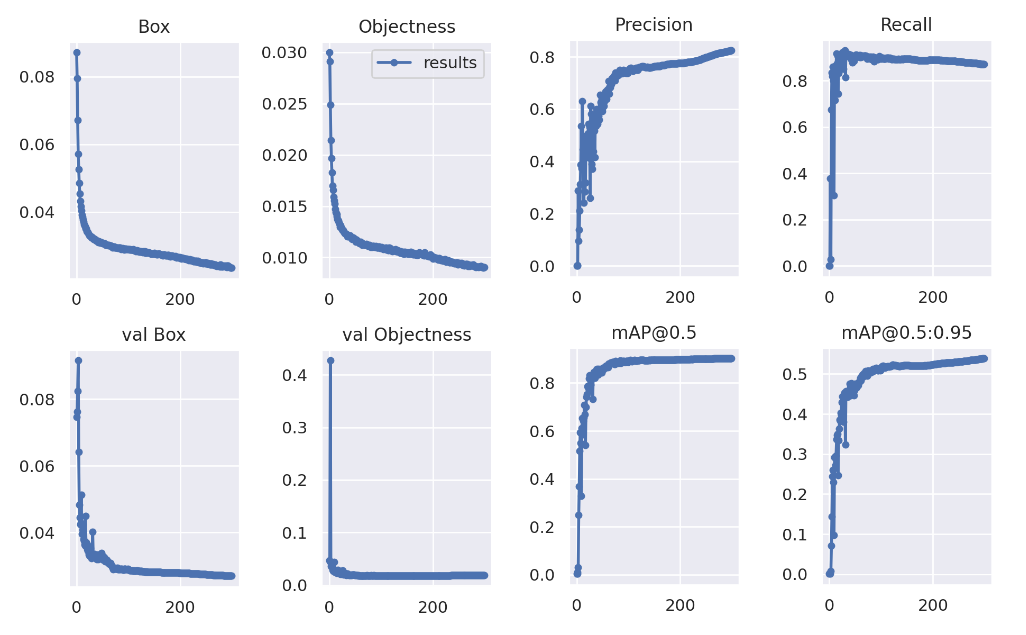


**Supplementary Figure 4.** Training process of machine learning

Before use, the images were preprocessed using nine steps; details of steps 1–9 are provided in Supplementary Text 1.


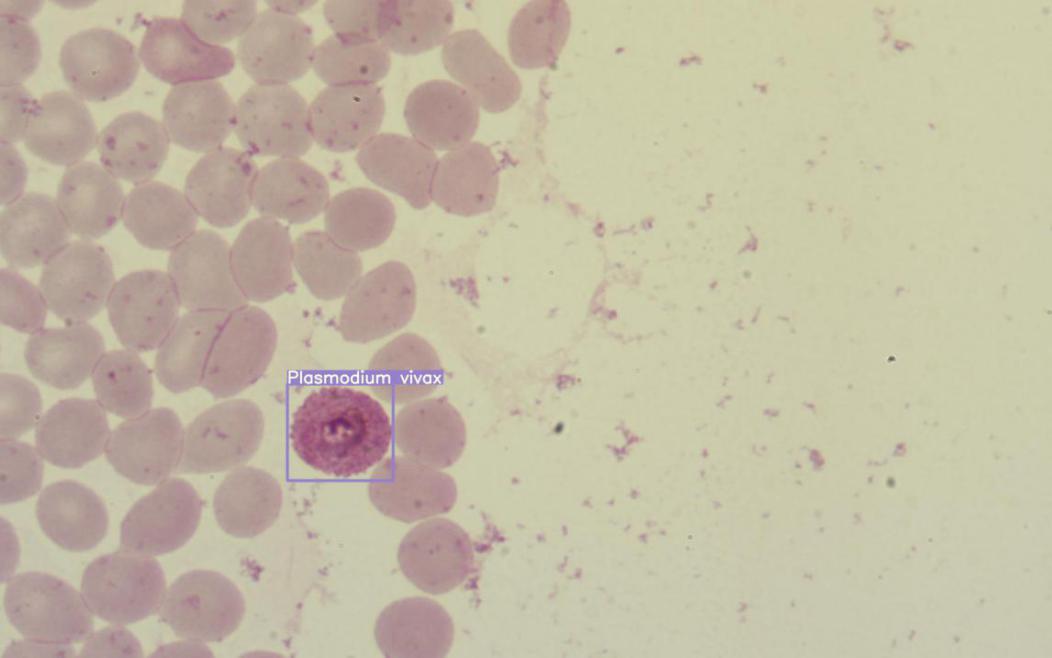


**Supplementary Figure 5.** Output results of *Plasmodium* classification, here *P. vivax* (*Plasmodium* parasites are marked using a box)

# Supplementary Text 1:

# Conventional nested PCR

# DNA was extracted from dried blood spots using the QIAamp 96 DNA Blood Kit from Qiagen, following the manufacturer's protocol. This kit is designed to efficiently extract DNA from blood samples.

# PCR Step 1:

# Template: 2 µL of the extracted DNA was used as the template in a 20-µL PCR reaction mixture.

# Reaction mixture: The PCR reaction mixture contained 0.25 mM dNTP, 10 mM Tris-HCL, 30 mM KCl, 1.5 mM MgCl2, and 1.0 unit of Taq polymerase. These components are necessary for DNA amplification.

# Primers: The primers used in this step were targeted to the 18S ribosomal RNA gene. Primers are short DNA sequences that bind to specific regions of the DNA and serve as starting points for DNA replication. Forward primer (*P. falciparum*): 5-CCGACTAGGTGTTGGATGAAA GTGTTAA-3; Forward primer (*P. vivax*): 5-CCGACTAGGCTTTGGATGAAAGATTTTA-3; Reverse primer: 5-AACCCAAAGACTTTGATTTCTCATAA-3; Taqman probe: 5-(Cy5)-AGCAATCTAAAAGTCACCTCGAAAGATGACT-BHQ-2-3; Taqman probe: 5-(TAMRA)-AGCAATCTAAGAATAAACTCCGAAGAGAAAATTCT-BHQ-2-3

# PCR cycle: The thermal cycling protocol consisted of the following steps:

# Initial denaturation at 95°C for 5 min: this step denatures the double-stranded DNA, separating it into two single strands.

# A total of 30 cycles of amplification, including the following steps:

# Denaturation at 95°C for 30 s: for further denaturation of the DNA strands.

# Annealing at 55°C for 1 min: to allow the primers to anneal (bind) to their complementary sequences in the DNA.

# Extension at 72°C for 2 min: the Taq polymerase synthesizes new DNA strands using the primers as a template.

# Final elongation step at 60°C for 10 min: this step allows any remaining incomplete DNA strands to be fully extended.

# PCR Step 2:

# Template: 2 µL of the first PCR product (amplified DNA from Step 1) was used as the template in a new 20-µL PCR reaction mixture.

# Reaction mixture: The PCR reaction mixture and components were the same as in Step 1.

# PCR cycle: The same thermal cycling protocol used in Step 1 was followed.

# Visualization of amplified products:

# Agarose gel electrophoresis: The amplified DNA products from both PCR steps were visualized using 2% agarose gels. Agarose gel electrophoresis is a common method to separate and visualize DNA fragments based on their size.

# Staining: The agarose gel was stained with ethidium bromide, a fluorescent dye that binds to DNA. Under UV light, the DNA bands become visible, allowing researchers to determine the presence and size of the amplified DNA fragments.

# 2 Multiplexed Real-Time PCR

RT-PCR conditions: The universal RT-PCR conditions consisted of an initial denaturation step at 95°C for 10 min, followed by a single cycle of annealing/extension at 60°C for 1 min. This was followed by 40 cycles of denaturation at 95°C for 15 s and annealing/extension at 60°C for 1 min.

Reaction mixture: The reaction mixture used in the RT-PCR had a total volume of 25 µL, comprising 5 µL of DNA, 12.5 µL of TaqMan universal master mix, and specific primers and probes.

Primers and probes: The forward primer used was named Plasmo2, a general primer for detecting *Plasmodium* species. Additionally, species-specific probes were used to distinguish between *P. falciparum* and *P. vivax*.

Fluorophores: Modifications were made to the fluorophores used in the probes. Each probe had a distinct fluorophore to allow specific detection of the targeted *Plasmodium* species.

Threshold values: The threshold values for detecting *P.* *falciparum* and *P.* *vivax* were set to 0.1 and 0.04 relative fluorescence units (RFU) at 40 cycles, respectively. These values were used to determine the minimum fluorescence level required for a sample to be considered positive for the respective *Plasmodium* species.

# Supplementary Text 2:

# Image Processing Steps

# Step 1:

Median filtering is a nonlinear signal processing technique based on ranking statistical theory that can effectively suppress noise. By replacing the value of a pixel point in a digital image with the median of the pixel points in the neighborhood set of that point, the value of the pixel points to be processed is approximated to the surrounding pixels. This eliminates isolated noise points (such as the pepper noise that may exist in a parasite image) and makes the image smooth and clear, which improves the following image processing operations. All input images must be denoised by the median filter to produce a clear image. The detailed process of median filtering is as follows:

1) Define sliding window: N*N.

2) Obtain the image size: H*W.

3) The initial window position is at the top left of the image.

4) Read the gray value in the window.

5) Sort the grayscale values in the window from largest to smallest.

6) Determine the median of the gray value of the window.

7) Copy the median to the center of the window.

8) Move the window to the right M positions and repeat steps 4–7 until the right side of the window is at the far-right side of the image.

9) Restore the window to the leftmost position and move down N positions; repeat step 8.

# Step 2:

An image flip operation is equivalent to an image mirroring operation, which can be divided into up/down and left/right mirroring. An image rotation operation will rotate the image clockwise or counterclockwise along the center of a certain angle. While the two operations are similar, the content of the image obtained differs. Both the image flip and rotation operations are designed to increase the number and diversity of images at the data level and reduce the possibility of model overfitting. Therefore, they can be used to improve the accuracy and robustness of the model. The detailed process of image flipping is as follows:

1) Obtain the image size H*H.

2) Transform the image into a two-dimensional matrix.

3) Retain the diagonal elements from the top left to the bottom right of the matrix and swap the pixel values with the diagonal pair of pixel points of the matrix.

# Step 3:

Random small adjustments to brightness, saturation, and contrast can simulate parasite data acquisition by representing the brightness and different contrast parameter settings of the microscope camera for different imaging results. Different degrees of staining (deep and shallow) can provide image color depth, which can enrich the diversity of data. The model requires accurate recognition of images under various illumination and color tones, and these data simulation steps at the image processing stage are useful to mitigate recognition problems in more complex scenes. The noted random method only adds perturbations within a tiny interval of brightness, contrast, and saturation adjustment parameters, and the processed image hue, saturation, and brightness are all within the interval, with no fixed target values. The saturation adjustment result affects the contrast result; therefore, adjustment of saturation alone is sufficient to accomplish all enhancement targets.

The detailed process of saturation adjustment is as follows:

1) Calculate the minimum and maximum value of the RGB component of the current pixel point.

2) Calculate the delta (difference between the two values / 255).

3) If delta = 0, go to the next pixel point to execute steps 2 and 3; if delta > 0, continue to step 4.

4) Calculate the value as the sum of two values / 255.

5) Transform the RGB space to HSL (Hue, S saturation, and L brightness) space. L = value / 2

6) If L < 0.5, then S = delta / value; otherwise, S = delta / (2 − value).

7) Apply custom saturation incrementation; increment / 100 is the saturation adjustment ratio. If the increment is > 0 to increase saturation, and vice versa to reduce saturation.

8) Based on different formulas for the new RGB value, generate a new image.

The detailed process of brightness adjustment is as follows:

1) Calculate the RGB pixel mean value of the image (M).

2) Remove the average value of each pixel of the image (M).

3) Multiply the pixel P by the contrast factor after removing the average value.

4) Multiply the pixel P + M by the luminance system.

5) Finish reassigning the RGB value to the pixel.

# Step 4:

The image is subjected to a median filtering operation, replacing itself with the average of the pixels around each pixel; the convolution kernel is 3 × 3.

# Step 5:

Add Gaussian white noise to the image. A noise with a probability density function obeying Gaussian distribution (i.e., normal distribution) is added to the original image to generate a new image.

# Step 6:

Mosaic data enhancement is a technique for image enhancement. Each large image is stitched into four images, in the order of one, two, three, and four quadrants, to obtain a large image, which is in turn used as the input for the model to detect. This method accelerates the training speed of the model, reduces the risk of overfitting the model, enriches the foreground and background information of the images, and improves the accuracy and robustness of the model.

# Step 7:

MixUp data enhancement is an image enhancement technique that enhances the richness and diversity of the data and the ability of the model to extract features related to the foreground. Two different images are weighted and added (e.g., 0.6 × pic1 + 0.4 × pic2) to obtain a new image containing the contents of both original images. The model output based on this image is expected to have the same weighted fusion as the true labels of the two images (e.g., 0.6 × label1 + 0.4 × label2). The model focuses more on the respective foreground information of each image and the corresponding scale in the learning process, thereby allowing it to learn the information related to the foreground and enhancing the recognition accuracy and generalization performance.

# Step 8:

Image cropping and scaling to 640 × 640 pixels.

# Step 9:

Image normalization. Pixel values were standardized to a normal distribution with a mean of 0 and variance of 1, and NumPy was converted to tensor vectors.

**Supplementary Table 1. Patients diagnosed with malaria according to causative species (*N* = 380)**

| *Plasmodium* species | Number of patients |
| --- | --- |
| *P. falciparum* | 32 |
| *P. vivax* | 331 |
| *P. malariae* | 8 |
| *P. ovale* | 7 |
| *P. knowlesi* | 1 |
| *P. cynomolgi* | 1 |
| Total | 380 |

**Supplementary Table 2.** Confusion matrix of *Plasmodium* detection

|  | Gold standard positive | Gold standard negative |
| --- | --- | --- |
| Algorithm recognizes positive | 1,018 | 55 |
| Algorithm recognizes negative | 43 | - |
